# PD70 A Mapping Study Of EORTC-QLQ-C30 To EQ-5D-5L Among Korean Patients With Non-Small Cell Lung Cancer

**DOI:** 10.1017/S0266462324003271

**Published:** 2025-01-07

**Authors:** Hyorim Lee, Jeonghoon Ahn, Hyerin Seo, Doyeon Jin

## Abstract

**Introduction:**

This study aimed to develop an algorithm to map the clinically popular European Organization for Research and Treatment of Cancer (EORTC) Quality of Life Questionnaire Core 30 (QLQ-C30) to the prevailing health technology assessment outcome measure, the EuroQol 5D-5L questionnaire (EQ-5D-5L), to investigate the quality of life in patients with non-small cell lung cancer (NSCLC).

**Methods:**

Mapping models were estimated using EQ-5D-5L, the EQ visual analogue scale (EQ-VAS), and the QLQ-C30 results from patients with NSCLC. This study used data at baseline from all three quality of life scores (n=289) to estimate the mapping algorithm. Linear regression was conducted on each domain of the QLQ-C30 as follows: physical functioning, global health status, emotional function, diarrhea, financial difficulties, insomnia, fatigue, and pain. Quality of life data collected at six (n=176) and 12 weeks (n=60) were used to evaluate the validity of the estimated mapping algorithm. The EQ-VAS was used to compare correlations with the algorithm.

**Results:**

The estimated algorithm had a root mean squared error of 0.0937 and an adjusted R-squared of 0.7227. The correlations between the estimated and the observed EQ-5D-5L values at baseline, six weeks, and 12 weeks were 0.8546, 0.8493, and 0.8846, respectively. The estimated and observed values had a high correlation. Also, the bias between the predicted and the observed EQ-5D-5L values at six and 12 weeks was 0.0113 and 0.0292, respectively. Hence, the mapping algorithm had a good predictive validity.

**Conclusions:**

The mapping algorithm can be used to convert a cancer-specific health-related quality of life measure to a preference-based health-related utility measure, which is useful in health technology assessment. Mapping EORTC-QLQ-C30 onto the EQ-5D-5L enables the estimated EQ-5D-5L scores of patients with cancer to be used in economic evaluations such as cost-utility analyses.

